# Measurement of the Robot Motor Capability of a Robot Motor System: A Fitts's-Law-Inspired Approach

**DOI:** 10.3390/s130708412

**Published:** 2013-07-02

**Authors:** Hsien-I Lin, C. S. George Lee

**Affiliations:** 1 Graduate Institute of Automation Technology, National Taipei University of Technology, Taipei 10608, Taiwan; 2 School of Electrical and Computer Engineering, Purdue University, West Lafayette, IN 47907, USA; E-Mail: csglee@purdue.edu

**Keywords:** robot motor capability, pseudo-index of motor performance, speed-accuracy constraint, Fitts's law

## Abstract

Robot motor capability is a crucial factor for a robot, because it affects how accurately and rapidly a robot can perform a motion to accomplish a task constrained by spatial and temporal conditions. In this paper, we propose and derive a pseudo-index of motor performance (*pI_p_*) to characterize robot motor capability with robot kinematics, dynamics and control taken into consideration. The proposed *pI_p_* provides a quantitative measure for a robot with revolute joints, which is inspired from an index of performance in Fitts's law of human skills. Computer simulations and experiments on a PUMA 560 industrial robot were conducted to validate the proposed *pI_p_* for performing a motion accurately and rapidly.

## 1. Introduction

Robots are widely-used in manufacturing tasks, such as assembly [1], cutting [2], deburring [3], *etc.* Without loss of generality, these manufacturing tasks can be decomposed into a sequence of motor tasks that are usually described by spatial and temporal constraints on objects in the environment [4–6]. Thus, a major problem for a robot to accomplish a task is to satisfy the spatial and temporal constraints of a task. A spatial-temporal constraint requires a robot to perform motor movements accurately in a desired trajectory task. Usually, a robot is asked to achieve a given position with a minimum movement time. However, each robot has its own motor capability according to its mechanical and electrical system and may not be qualified for tasks with strict spatial-temporal constraints.

Robot motor capability is the motor ability for a robot to accomplish a task constrained by spatial and temporal conditions. Traditionally, a robot was evaluated by its accuracy and repeatability [7–10]. American National Standard defined the static and dynamic performance characteristics for industrial robots and robot systems [11,12]. Throughout the previous work, there has not been a performance metric to measure the robot motor capability of a robot motor system for tasks constrained by spatial and temporal conditions. Thus, there is a need to develop a quantitative measure of a robot motor system.

In this paper, the proposed method for characterizing the motor capability of a robot is inspired by Fitts's law, which is one of the well-known metrics for studying human rapid movements. Fitts's law was revealed by Paul Fitts in 1954, showing the information capacity of a human motor system. In Fitts's law, the motor performance is the ability to consistently produce movements and is described by two main factors—speed and accuracy. Fitts obtained the following equation, often called Fitts's law, as:

(1) $$T_{\text{mt}}=a+b\cdot \log_{2}(\frac{2D}{\text{w}})$$

where *T_mt_* is the minimum movement time and *a* and *b* are proportional constants, and Fitts quantitatively defined an index of task difficulty (*I_d_*) as $\log_{2}\left(\frac{2D}{W}\right)$ (bits/response) to specify the minimum required information for rapidly-aimed movements to accomplish a given task, where D is a target distance and W is a target width defined as the allowable tolerance along the moving direction. Fitts also defined an index of performance, *I_p_*, to indicate the fixed information capacity:

(2) $$I_{p}=\frac{1}{T_{\text{mt}}}\cdot \log_{2}(\frac{2D}{W})$$

and found that *I_p_* is relatively constant over a considerable range of movement amplitudes and tolerance limits.

Actually, Fitts's equation is a special case of the quasi-power function with a varying exponent, *n*, as *n***→ ∞**[13]. The quasi-power function with exponent ***n*** is shown as $T_{mt}=a_{n}+b_{n}\cdot {\left(\frac{D}{W}\right)}^{\frac{1}{n}}$. When *n***→** 1, the quasi-power function becomes a linear function as:

(3) $$T_{mt}=a_{1}+b_{1}\cdot (\frac{D}{W})$$

Equation (3) is re-written as a linear speed-accuracy relationship:

(4) $$W=a^{\prime }+b^{\prime }\cdot (\frac{D}{T_{mt}})$$

where *a'* and *b'* are proportional constants, and the index of performance (*I_p_*) of Fitts s law in Equation (3) is defined as $\frac{1}{b_{1}}$ by ignoring the constant, *α*_1_ Thus, $\frac{1}{b_{1}}$ in Equation (4) indicates the index of performance, as well, because smaller *b*_1_ results in smaller *T_mt_* and smaller *b'* if *D* and *W* are fixed in both equations.

The paper proposes the method to derive the speed-accuracy constraint of a robot system the same as Equation (4), and therefore, the pseudo-index of motor performance (*pI_p_*) of the robot system is defined as $\frac{1}{b^{\prime }}$ in Equation (4). The speed-accuracy constraint is characterized with robot kinematics, dynamics and control taken into consideration. Thus, the mathematical derivations of *pI_p_* are applicable to other robots with revolute joints. Furthermore, *pI_p_* is a quantitative measure for the motor capability of a robot. The basic concept of our approach is to treat a robot motor system as an information channel and determine the robot motor capability. Since the information capability of a channel is affected by the inaccuracy of kinematics and dynamics models, we can quantitatively measure it by the variability of movements that a robot aims to produce. To verify the proposed method, computer simulations and experiments on PUMA 560 and 260 industrial robots are conducted to verify the validity of the proposed approach in understanding the motor capability of a robot motor system.

This paper is organized as follows. In Section II, we formulate a robot task as an optimization problem constrained by the proposed speed-accuracy constraint derived from the kinematics, dynamics and control of a robot motor system. In Section III, we present the proposed pseudo-index of motor performance (*pI_p_*) derived from the speed-accuracy constraint and show how *pI_p_* is utilized to evaluate the motor capability of a robot. In Section IV, computer simulations and experiments on PUMA 560 and 260 industrial robots are presented. Discussions and conclusions are summarized in Section V.

## 2. Speed-Accuracy Constrained Optimization

### 2.1. Problem Formulation

We formulate a robot task as an optimization problem subject to spatial and temporal constraints. The proposed speed-accuracy constraint characterizes the relationship between the robot joint speed and the Cartesian position error of the end-effector of the robot. By utilizing the proposed speed-accuracy constraint, the optimization problem is solved without violating the robot motor capability. We first formulate a basic rapid robot movement that moves its end-effector to reach a desired joint location, ***θ*** = [*θ*_1_, *θ_2_*,…, *θ_i_*,…, *θ_n_*]*^T^*, with an allowable tolerance, *ε_x_, ε_y_* and *ε_z_* (*i.e.*, spatial constraint), in the Cartesian space by the maximal linear speed (*i.e.*, temporal constraint), where *N* is the number of degrees of freedom (DOF) and the superscript, *T*, denotes a matrix transpose. Here, we need to determine the joint velocities that introduce the maximal linear speed of the end-effector. The joint velocities are represented by a vector as ***θ̇*** = [*θ̇*_1_, *θ̇*_2_, …, *θ̇_i_*,… , *θ* ˙*_n_*]*^T^*, and the basic rapid movement of the end-effector of a robot is formulated as an optimization problem as:

(5) $$\begin{array}{c} \max{\left\|J_{d}(\theta )\dot{\theta }\right\|}_{2}\\ \text{subject to}\left|d_{x}\right|\leq \epsilon_{x},\left|d_{y}\right|\leq \epsilon_{y,}\left|d_{z}\right|\leq \epsilon_{z},\text{and}\\ \;0\leq \left|{\dot{\theta }}_{i}\right|\leq {\dot{\theta }}_{i}^{\max},\text{for}\;i=1\;\mathrm{to}\;N \end{array}$$

where ‖ · ‖_2_ is the Euclidean-norm operation, ***J****_d_*(***θ***) is the 3xN Jacobian matrix relating the joint velocity to the Cartesian velocity of the end-effector, *d_x_, d_y_* and *d_z_* are the respective Cartesian position errors, *ε_x_, ε_y_* and *ε_z_* ≥ 0 are the allowable position errors and ${\dot{\theta }}_{i}^{\text{max}}\geq 0$ is the maximum joint, *i*, velocity that can be driven. Obviously, ***θ̇****^max^* in Equation (5) may not be the optimal solution to the problem, because it may violate the spatial constraint described by *ε_x_, ε_y_* and *ε_z_*. Thus, both the spatial and temporal constraints must be satisfied simultaneously in this optimization. To consider these constraints, an appropriate speed-accuracy constraint must be derived.

### 2.2. Robot Speed-Accuracy Constraint

Since the spatial inaccuracy of the end-effector of a robot with revolute joints is caused by the inaccuracy of robot kinematics and dynamics models and the disturbance of the environment [8], we represent the Cartesian position error of the end-effector of an *N*-DOF manipulator (e.g., a PUMA robot) with revolute joints by a linear model expressed as:

(6) $$\begin{array}{ll} \left[\begin{array}{c} {\text{d}}_{x}\\ d_{y}\\ d_{z} \end{array}\right] & =[J_{k}^{d}(\theta )\Delta d+J_{k}^{\text{a}}(\theta )\Delta a+J_{k}^{\alpha }(\theta )\Delta \alpha +J_{k}^{\beta }(\theta )\Delta \beta ]+J_{d}(\theta )\Delta \theta \\ & =C(\theta )+J_{d}(\theta )\Delta \theta \end{array}$$

where *d_x_, d_y_* and *d_z_* are the respective Cartesian position errors along each coordinate axis, $J_{k}^{d}(\theta )$, $J_{k}^{a}(\theta )$, $J_{k}^{\alpha }(\theta )$ and $J_{k}^{\beta }(\theta )$ are the 3 x N kinematic-error matrices [8], ***J****_d_*(***θ***) is the 3 x N Jacobian matrix, ***θ*** is an *N* x 1 joint position vector, (d, a, *α, β*) are the D-Hparameters [14] in an *N* x 1 vector form, (Δd, Δa, Δ*α, * Δ*β*) denote small changes in the corresponding parameters and ***C***(***θ***) is a 3 x 1 vector, summing all the kinematic errors, due to the D-H parameters (d, a, *α, β*); this is due to the mechanical tolerance in manufacturing the links and joints of the robot.

**Figure 1.**
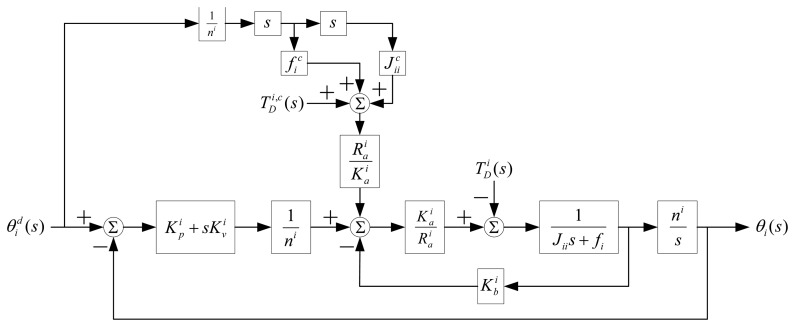
Proportional-plus-derivative (PD) control scheme of a robot joint, *i*.

To derive Δ***θ*** in Equation (6), we assume that the widely-used “computed torque” technique with a proportional-plus-derivative (PD) controller [15] is used. Thus, each robot joint is modeled as a single-input-single-output system with disturbance from other joints. The coupling effects from other links are considered as a disturbance to the robot. A PD control is utilized to servo the manipulator system to track the desired trajectory, and the feedforward torques along the actual trajectory are computed to compensate for the nonlinear disturbance. A block diagram showing the PD control scheme for joint, *i*, is shown in Figure 1.

In Figure 1, $J_{ij}^{c}(\theta )$, $f_{i}^{c}$, $G_{i}^{c}(\theta )$ and $V_{i}^{c}$ are, respectively, the computed counterparts of the actual effective inertia terms, *J_ij_*(***θ***), effective damping coefficient, f_i_, gravity terms, *G_i_*(***θ***), and Coulomb friction, *V_i_*, at the actual shaft of joint, *i*, of the manipulator. *n^i^* is the gear ratio, armature resistance, $R_{a}^{i}$, motor constant, $K_{a}^{i}$, and the back electromagnetic force constant, $K_{b}^{i}$, is known dc motor parameters for joint, *i*. $K_{p}^{i}$ and $K_{\upsilon }^{i}$ are, respectively, position and velocity feedback gains of the controller. Their values are chosen subject to the mechanical resonant frequency constraint of the manipulator. $\theta_{i}^{d}(s)$ and *θ_i_*(*s*) are, respectively, the desired and actual angular displacement of joint, *i*. $T_{D}^{i}(s)$ is the disturbance in Laplace transform and $T_{D}^{i,c}(s)$ is the computed feedforward torque compensating for the disturbance.

In order not to excite the mechanical resonant frequency of the manipulator, the undamped natural frequency is set to no more than one-half of the structure resonant frequency. Complying to this constraint, we can obtain the following relation and the upper bound of $K_{p}^{i}:$

(7) $$0\leq K_{p}^{i}\leq \frac{J_{0}^{i}{\left(\omega_{0}^{i}\right)}^{2}R_{a}^{i}}{4K_{a}^{i}}$$

where $\omega_{0}^{i}$ and $J_{0}^{i}$ are, respectively, the measured structural resonant frequency and inertia of joint, *i*, at a known location. Since Figure 1 is a second-order system, the damping coefficient, ζ*_i_*, of the system can be obtained as:

(8) $$\zeta_{i}=\frac{R_{a}^{i}f_{i}+K_{a}^{i}K_{b}^{i}+K_{a}^{i}K_{\upsilon }^{i}}{\sqrt[{2}]{K_{p}^{i}K_{a}^{i}R_{a}^{i}J_{ii}}}$$

In order to avoid the oscillatory underdamped response, ζ*_i_* is set to ≥1. Thus, $K_{\upsilon }^{i}$ becomes:

(9) $$K_{\upsilon }^{i}\geq \frac{\sqrt[{2}]{K_{a}^{i}K_{p}^{i}R_{a}^{i}J_{ii}}-R_{a}^{i}f_{i}-K_{a}^{i}K_{b}^{i}}{K_{a}^{i}}$$

Because the actual *J_ii_* and *f_i_* are usually not available, we use their computed counterparts, $J_{ii}^{c}$ and $f_{i}^{c}$, from the manipulator dynamic model. Substituting Equation (7) into Equation (10), we obtain $K_{\upsilon }^{i}$ as:

(10) $$K_{\upsilon }^{i}\frac{R_{a}^{i}\omega_{0}\sqrt{J_{0}^{i}J_{ii}^{c}}-R_{a}^{i}f_{i}^{c}-K_{a}^{i}K_{b}^{i}}{K_{a}^{i}}$$

To determine Δ***θ*** in Equation (6), we utilize Figure 1 to evaluate the steady-state error of *θ* by a velocity-related joint position command, *θ_d_*(*t*) = *At*, where *A* is the amplitude of the input. The steady-state error of *θi* is derived as:

(11) $$\begin{array}{ll} \Delta \theta_{i} & =\frac{n^{i}R_{a}^{i}}{K_{a}^{i}K_{p}^{i}}\left(\sum_{j}\sum_{k}\Delta {\text{H}}_{\text{ijk}}(\theta ){\dot{\theta }}_{j}{\dot{\theta }}_{k}+\Delta G_{i}(\theta )+\Delta V_{i}\right)\\ & =\frac{4n^{i}}{J_{0}^{i}{\left(\omega_{0}^{i}\right)}^{2}}\left({\dot{\theta }}^{T}\Delta H_{i}(\theta )\dot{\theta }+\Delta G_{i}(\theta )+\Delta V_{i}\right) \end{array}$$

where $\Delta H_{\text{ijk}}(\theta )=H_{\text{ijk}}(\theta )-H_{\text{ijk}}^{c}(\theta )$, $\Delta G_{i}(\theta )=G_{i}(\theta )-G_{i}^{c}(\theta )$, $\Delta V_{i}=V_{i}-V_{i}^{c}$and Δ***H****_i_*(***θ***) is an *N* x *N* symmetric matrix, whose elements are Δ*H_ijk_* (***θ***), where *j* and *k* are integers and 1 ≤ j ≤ *N* and 1 ≤ *k* ≤ *N*.

Replacing the elements of Δ***θ*** in Equation (6) with Δ*θ_i_* in Equation (11), we obtain the Cartesian position errors of the end-effector of the robot in the form of speed-accuracy constraint as:

(12) $$\left[\begin{array}{c} d_{x}\\ d_{y}\\ d_{z} \end{array}\right]=C(\theta )+J_{d}(\theta )K\Delta P(\theta )+J_{d}(\theta )K{\dot{\Phi }}^{T}\Delta H(\theta )\dot{\Theta }$$

where Δ***P***(***θ***) is an *N* x 1 vector and Δ***P***(***θ***) = [Δ*G_1_*(***θ***) + Δ*V_1_*,…, Δ*G_N_*(***θ***) + Δ*V_N_*] = Δ*G*(***θ***)+Δ*V*, where Δ*G* = [Δ*G*_1_(***θ***),…, Δ*G_N_*(***θ***)]*^T^* and Δ***V*** = [Δ*V*_1_,…, Δ*V_N_*]*^T^*,***K****is* an *N* × *N* diagonal matrix, whose elements are $\frac{4n^{1}}{J_{0}^{1}{\left(\omega_{0}^{1}\right)}^{2}}$,…, $\frac{4n^{N}}{J_{0}^{N}{\left(\omega_{0}^{N}\right)}^{2}}$, Δ***H***(***θ***) is an *N*^2^ x *N*^2^ matrix, and Δ***H***(***θ***) = diag{Δ***H***_1_(***θ***),… Δ***H****_n_*(***θ***)}, **Φ̇** is an *N*^2^ x *N* matrix and $\dot{\Phi }=[{\dot{\theta }}_{1}^{\prime },0,\cdots ,0;0,{\dot{\theta }}_{2}^{'},0,\cdots 0;\cdots ;0,\cdots 0,{\dot{\theta }}_{N}^{'}]$, where ${\dot{\theta }}_{i}^{\prime }=\dot{\theta }$ for *i* = 1 to *N*, and **Θ̇** is an *N*^2^ x 1 matrix and $\dot{\Theta }={\left[{\dot{\theta }}_{1}^{"}\cdots {\dot{\theta }}_{N}^{"}\right]}^{T}$, where ${\dot{\theta }}_{i}^{"}={\dot{\theta }}^{T}$ for *i* = 1 to *N*.

From Equation (12), it shows that when the joint speed increases (***θ̇****_new_* = *κ****θ̇****_old_, κ* ≥ 1) and the desired joint positions, *θ*, are fixed, a larger absolute Cartesian position error (|*d_x_*|, |*d_y_*| and |d_z_|) will be generated. Thus, Equation (12) indicates the trade-off relationship between the spatial accuracy and the joint speed.

## 3. Pseudo-Index of Performance for a Robot System

The purpose of determining the pseudo-index of the performance, ***pI****_p_*, of a robot motor system is to obtain a quantitative measure. After the speed-accuracy constraint is obtained, we can derive and calculate the index of the performance, ***pI****_p_*, of a robot motor system from this constraint. Thus, we define the pseudo-index of the performance (*pI_p_*) of the linear speed-accuracy equation in Equation (4) as $pI_{p}≜\frac{1}{b^{\prime }}$ to quantitatively indicate the amount of information capacity of a robot motor system. According to the speed-accuracy constraint (see Equation (12)), ***C***(***θ***) + ***J****_d_*(***θ***)***K***Δ***P***(***θ***) can be referred to as *a'* in Equation (4). The joint velocity command is expressed as ***θ̇***_d_ = *ρ****θ̇***_0_, where ***θ̇****0* is a reference joint velocity vector and *ρ* > 0 is a scale factor to change the joint velocities. We can express Equation (12) in the form of Equation (4) as:

(13) $$\left[\begin{array}{c} d_{x}\\ d_{y}\\ d_{z} \end{array}\right]=[C(\theta )+J_{d}(\theta )K\Delta P(\theta )]+\left[\begin{array}{c} \frac{1}{pI_{px}}\\ \frac{1}{pI_{py}}\\ \frac{1}{pI_{pz}} \end{array}\right].\rho^{2}$$

where *pI_px_, pI_py_* and *pI_pz_* are the pseudo-indices of performance on the *x*-, *y*- and z-axis of the Cartesian space, respectively, and are derived as:

(14) $$\begin{array}{c} pI_{px}={\left(I_{x}^{T}J_{d}\;(\theta )\;K{\dot{\Phi }}_{0}^{T}\Delta \text{H}\;(\theta )\;{\dot{\Theta }}_{0}\right)}^{-1}\\ pI_{py}={\left(I_{y}^{T}J_{d}(\theta )K{\dot{\Phi }}_{0}^{T}\Delta H(\theta ){\dot{\Theta }}_{0}\right)}^{-1}\\ pI_{pz}={\left(I_{z}^{T}J_{d}(\theta )K{\dot{\Phi }}_{0}^{T}\Delta H(\theta ){\dot{\Theta }}_{0}\right)}^{-1} \end{array}$$

where ***I****_x_*,***I****_y_* and ***I****_z_* are the unit vectors along the *x-, y*- and z-axis, respectively, Φ̇_0_ is an N^2^ x N matrix and $\dot{\Phi }=[{\dot{\theta }}_{1}^{\prime },0,\cdots ,0;0,{\dot{\theta }}_{2}^{'},0,\cdots 0;\cdots ;0,\cdots 0,{\dot{\theta }}_{N}^{'}]$, where ${\dot{\theta }}_{i}^{"}={\dot{\theta }}_{0}$for *i* = 1 to *N*, and **Θ̇**_0_ is an N^2^ x 1 matrix and $\dot{\Theta }={\left[{\dot{\theta }}_{1}^{"}\cdots {\dot{\theta }}_{N}^{"}\right]}^{T}$, where ${\dot{\theta }}_{i}^{"}={\dot{\theta }}_{0}^{T}$ for *i* = 1 to *N*.

**Figure 2.**
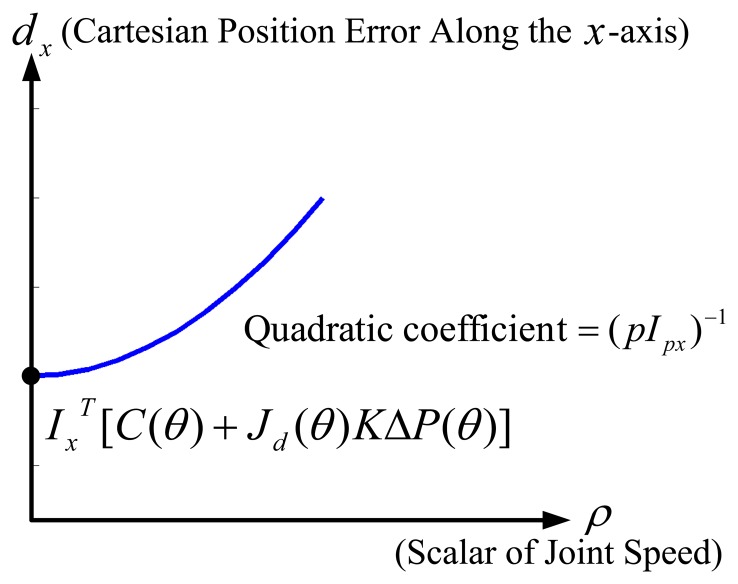
Quadratic function of the Cartesian position error of the end-effector of a robot along the *x*-axis (*d_x_*) with respect to the scalar of the joint velocity (*ρ*), where the quadratic coefficient is (*pI_px_*)*^-^*^1^ and *θ̇_d_* = *ρθ̇*_0_.

From Equation (13), we obtain the quadratic function of the Cartesian position errors, (*d_x_, d_y_, d_z_*), with respect to the scalar of the joint velocity, *ρ*.Figure 2 illustrates the obtained quadratic function of the Cartesian position error of the end-effector of a robot along the x-axis (*d_x_*) with respect to the scalar of the joint speed (*ρ*), where the intercept on the axis of the Cartesian position error of the end-effector of a robot is ***I****_x_^T^*[***C***(***θ***) + ***J****_d_*(***θ***)***K***Δ***P***(***θ***)]. From Figure 2 and Equation (13), it is shown that a robot is capable of performing more accurately with a larger *pI_p_* when the joint velocities, ***θ̇****_d_*, are fixed (*i.e., ρ* is fixed). In other words, when the Cartesian position tolerance, e, of a given task is fixed (*i.e., d_x_* is fixed), a robot with a larger *pI_p_* is capable of accomplishing the task with faster joint velocities. Interestingly, the pseudo-index of performance is advantageous to show the trade-off relationship between speed and accuracy, if we assume that there exists a fixed amount of information capacity of a robot motor system.

## 4. Computer Simulations & Experimental Work

Computer simulations were performed by MATLAB Toolbox [16], and experiments were performed on 6-degree-of-freedom PUMA 560 and 260 robots [14] with revolute joints to validate the proposed speed-accuracy optimization and demonstrate that the motor capability can be measured by the proposed pseudo-index of performance (*pI_p_*) on a rapid-moving task. In the experiments, the Cartesian position error was with respect to the 2D space, because we followed Fitt's experiments, where the position error was only measured in the 2D space. The Cartesian position error on the last dimension (z-axis) would not affect that on the other two dimensions (x- and y-axis), because they are independent.

### 4.1. Simulation on a PUMA 560 Robot

**Figure 3.**
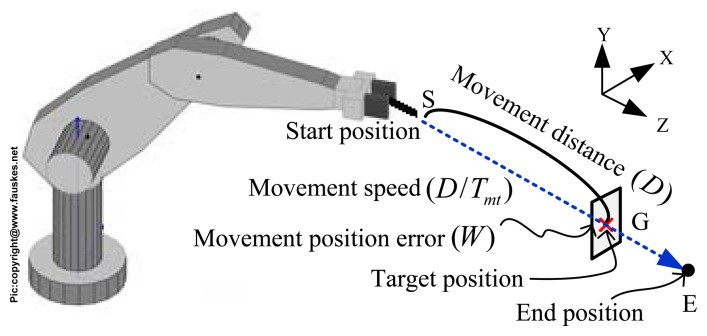
Scheme of our task, where the moving position error is defined as the error between the actual and target positions. (S: start position, G: target position and E: end position).

Figure 3 shows the scheme of our task. The robot was asked to move its end-effector to hit a target position (*G*) and stop at an end position (*E*) by various configurations of movement speed (*D/T_mt_*) and distance (*D*). With each configuration of movement speed and distance, this resulted in a Cartesian position error (*W*) at the end-effector.

In this simulation, a PUMA 560 industrial robot was asked to move with the maximum speed to hit the target position within the spatial tolerances, *σ_x_* and *σ_y_*, which denote the maximum position tolerances along the *x*- and y-axis, respectively. For simplicity, the robot only used joints 1, 2, and 3 to perform the task. Based on the task description, we formulated the task as an optimization problem subject to the spatial-accuracy constraints for maximizing the robot speed:

(15) $$\begin{array}{l} \max{\left\|J_{d}(\theta )\dot{\theta }\right\|}_{2}\\ \text{subject to}\left|d_{x}\right|\leq \sigma_{x,}\left|d_{y}\right|\leq \sigma_{y,}\;\text{and}\;0\leq \left|{\dot{\theta }}_{i}\right|\leq {\dot{\theta }}_{i}^{\max} \end{array}$$

where ***J****_d_*(***θ***) is the Jacobian matrix, *d_x_* and *d_y_* are the Cartesian position errors of the end-effector of the robot along the *x*- and *y*-axis, and ${\dot{\theta }}_{i}^{\text{max}}$ is the maximal velocity of joint, *i* (*i* = 1, 2, 3), that can be driven. The parameters of the PUMA 560 robot were from [17]. We set the distance from the tip of the pin to the coordinate frame of the sixth joint to 0.1 m.

First, we evaluated how the Cartesian position error was affected by Δ***m***, Δ***G***(***θ***) and Δ***V***(***θ***) under a fixed joint distance. We used joint-interpolated motion planning for the robot. Simply, we subtracted *C*(*θ*) from the Cartesian position errors of the end-effector of the robot. The joint distance, *θ_diff_*, between the initial and target joint positions was [π/4,π/4, -π /4, 0, 0, 0], and the execution time for all the joints was set to *T_mt_*. Thus, the joint velocities were represented as *θ_diff_/T_mt_* (rad/sec). If we set ***θ̇*** as the nominal joint velocities finishing *θ_diff_* by one second, the joint velocities were expressed as *ρ* ·; ***θ̇***, where *ρ* = 1*/* |*T_mt_*|. The joint velocity, acceleration and the inverse dynamics of the robot were calculated by the recursive Newton-Euler equations [14,18], and the Cartesian position error of the end-effector of the robot was calculated by ***J****_d_*(***θ***)Δ*θ* and verified by Equation (12).

**Figure 4.**
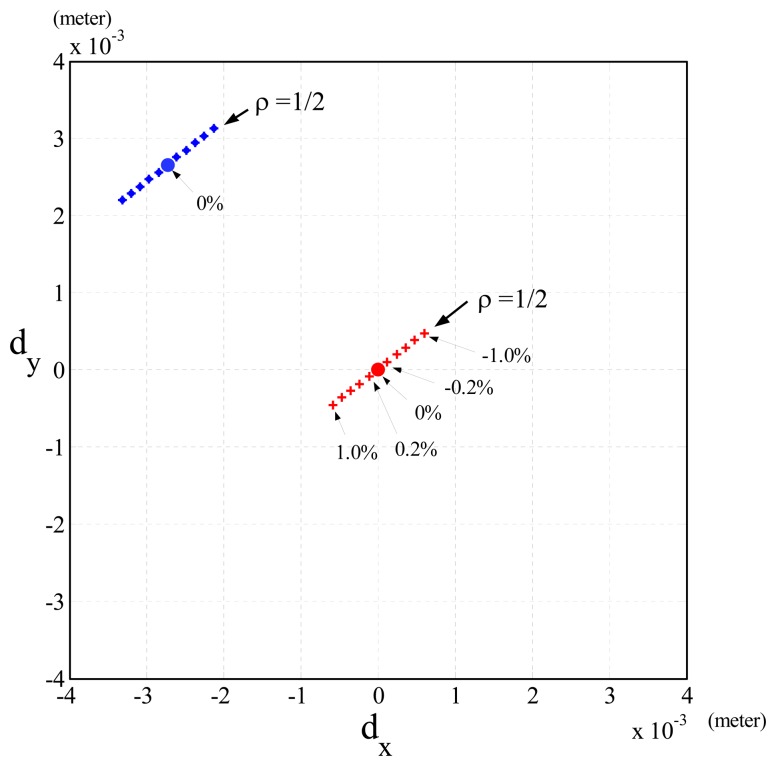
Simulation results of the Cartesian position errors of the end-effector of a PUMA 560 robot. (a) Case 1: Δ*V* = 0 and Δ*G*(*θ*) = 0 (red dot); (b) Case 2: AV = 0 and Δ*G*(*θ*) = 0 (blue dot); (c) Case 3: Δ*V* = 0, Δ*G*(*θ*) ≠ 0 and *ρ* = 0 (red crosses); (d) Case 4: Δ*V* ≠ 0, Δ*G*(*θ*) ≠ 0 and *ρ* = 0 (blue crosses).

**Figure 5.**
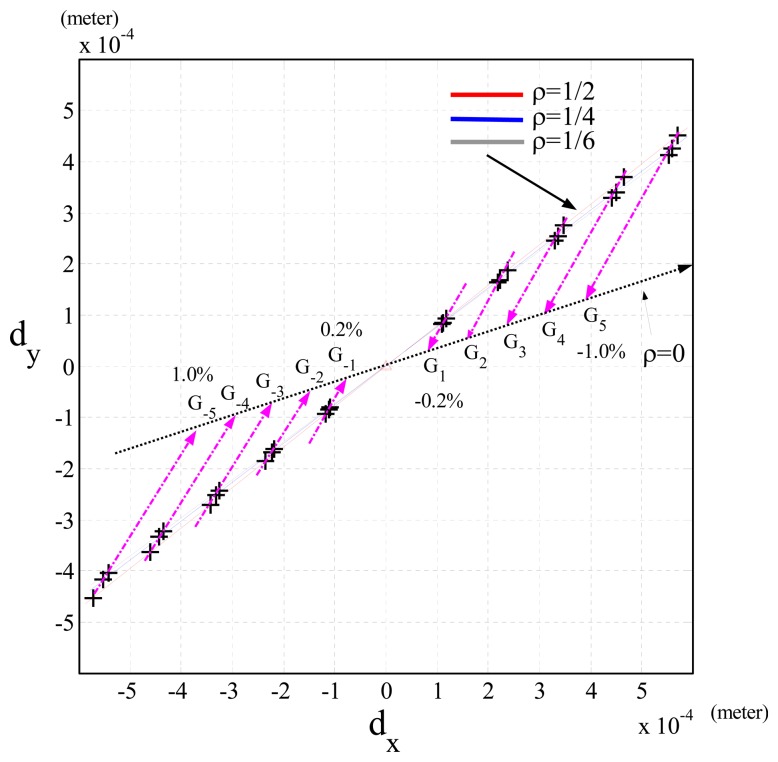
Simulation results of the Cartesian position errors of the end-effector of a PUMA 560 robot in Case 3 when the joint velocities are non-zero: Δ*V* = 0, Δ*G*(*θ*) = 0 and *ρ* ≠ 0 (crosses).

When the errors between the computed and actual masses, $\Delta m=\frac{m-m^{c}}{m}$, as (±0.1%), (±0.2%),… and (±1%), were assumed as the cause of the Cartesian position errors, we simulated four cases of a combination of Δ***V*** (Coulomb friction) and Δ***G***(***θ***) (errors between the actual and computed gravity terms). Their results are explained and discussed as follows:

Case (1) Δ***V*** = 0 and Δ***G***(***θ***) = 0: In this case, the simulation results showed that the Cartesian position errors of the end-effector of the robot (*d_x_* = *d_y_* = *d_z_* = 0) almost do not exist. Apparently, they can be validated by Equation (12), because of Δ*P*(***θ***) = 0 (Δ***V*** = 0, Δ***G***(***θ***) = 0 and Δ***H***(***θ***) = 0).
Case (2) Δ***V***≠ 0 and Δ***G***(***θ***) = 0: In this case, the simulation results of the Cartesian position errors along the *x*- and *y*-axis (*d_x_* and *d_y_*) were shown by the blue dots with Δ*m* = 0 in Figure 4. The Cartesian position errors were only due to Δ***V*** of Equation (12).
Case (3) Δ***V*** = 0 and Δ***G***(***θ***) ≠ 0: In this case, the simulation results showed that when *ρ* = 1/2 and Δ*m* was changed from 1.0% to -1.0%, the Cartesian position errors were shown by the line of red crosses in Figure 4 and caused by Δ***G***(***θ***) and Δ***H***(***θ***) of Equation (12). On the other hand, when the joint velocities were changed by *ρ* (1/2, 1/4, 1/6), Figure 5 shows that the slope of the line showing the Cartesian position errors was also changed. It shows that as *ρ*= 1/6 (gray line, lowest speed), the Cartesian position errors were least (highest accuracy). In addition, when Δ*m* was fixed, Figure 5 shows that the Cartesian position errors caused by different *ρ* were in a linear line (*G_i_* pink line), and the increment of the Cartesian position errors was proportional to *ρ*^2^, because ***J****_d_*(***θ***)***K*Φ̇***^T^* Δ***H***(***θ***)**Θ̇** in Equation (12) indicates that the Cartesian position errors were proportional to (***θ̇***^2^) (*i.e., ρ*^2^) when Δ*m* was fixed. However, none of the pink lines in Figure 5 did passed through the origin, but a point *G_i_*, (*i* = …, −2, −1,1, 2, …). These results were clearly shown by Equation (12), because the Cartesian position errors resulted from Δ***G***(***θ***) and Δ*H*(***θ***), but when the joint velocities were set to zero, the Cartesian position errors were only due to Δ***G***(***θ***) (*i.e., Gi*).
Case (4) Δ***V***≠ 0 and Δ***G***(***θ***) ≠ 0: This case has the same results as Case 3, except the lines showing the Cartesian position errors caused by fixed *ρ* did not pass through the origin.

Then, we evaluated the *pI_p_* of a PUMA 560 robot with various Δ*m's* and Cartesian distances, where Δ*m* = *m − m^c^, m* were the actual masses and *m^c^* were the computed masses. In other words, a robot with a different Δ*m* had a different motor capability, because Equation (14) shows that the pseudo-index of performance is affected by Δ***H***(***θ***) (*i.e.*, Δ*m*). We set the Cartesian distances between the start (*S*) and the target (*G*) positions to 0.0508, 0.1016, 0.2032 and 0.4064 m. The four movement velocities (*D/T_mt_* m/sec) for a specified distance (*D*) were set in Table 1. To investigate the Cartesian position errors of the end-effector of the PUMA 560 robot caused by the changes of the joint velocities, we subtracted the velocity-independent terms, ***C***(***θ***) + ***J****_d_*(***θ***)***K***Δ***P***(***θ***), from the Cartesian position errors at the target position. For *D* = 0.0508, 0.1016, 0.2032 and 0.4064 (m), the joint velocities were represented as $(\theta_{diff}^{D})/T_{mt}=\rho \cdot {\dot{\theta }}_{0}$, where *θ̇*_0_ is the nominal joint velocities finishing $\theta_{diff}^{D}$ by one second, *ρ* is a scalar and its value is *ρ* = 1*/* |*T_mt_*|. We changed the joint velocities by changing *ρ* with a 0.01 incremental value. The joint velocity, joint acceleration and the inverse dynamics of the robot were calculated by the recursive Newton-Euler equations [14,18], and the Cartesian position error of the end-effector of the robot was calculated by ***J****_d_*(***θ***)Δ***θ***.

**Figure 6.**
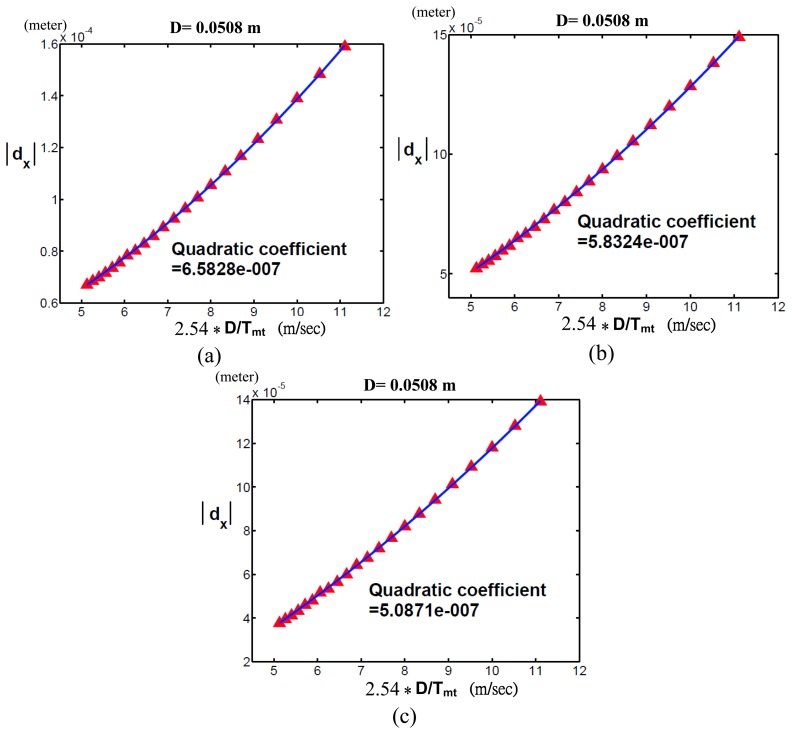
Simulation results of the Cartesian position errors of the end-effector of a PUMA 560 robot along the x-axis (*d_x_*) with respect to the Cartesian velocities (*D/T_mt_*) when *D* = 0.0508 and Δ*m* was (**a**) −1%; (**b**) −0.6%; (**c**) −0.2%. Red triangles represent the simulation results; the blue curve represents curve fitting.

Figure 6 shows the Cartesian position errors along the *x*-axis (*d_x_*) with respect to *D/T_mt_* when *D* = 0.0508 and Δ*m* was (a)−1%, (b)−0.6% and (c)−0.2%, respectively. In each sub-figure, we used a quadratic function to do curve fitting for our simulation results. Since the quadratic coefficient is the reciprocal of *pI_px_*, by referring to Figure 2, we noticed that Figure 6(c) had the greatest *pI_px_* among Figure 6(a-c). It was explained by the reason that Figure 6(c) had the least amount of Δm = −0.2%.

**Figure 7.**
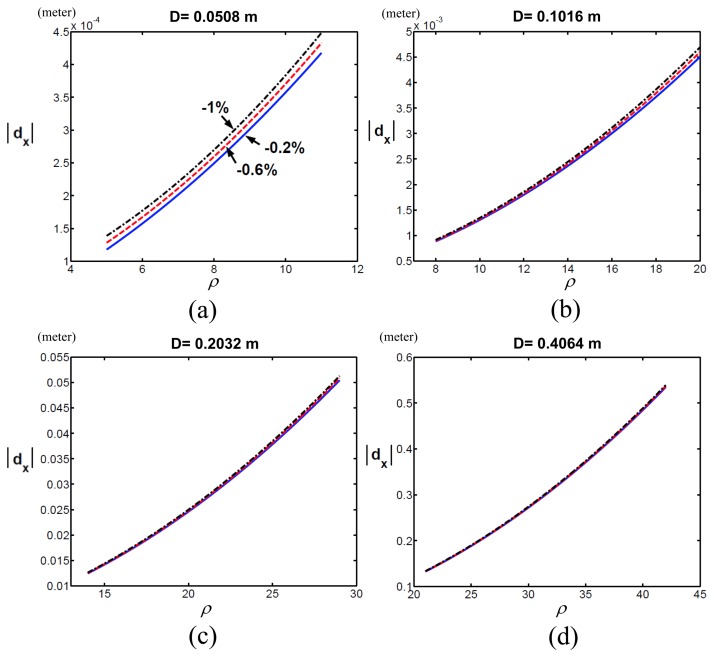
Simulation results of the Cartesian position errors of the end-effector of a PUMA 560 robot along the x-axis (*d_x_*) with respect to the scalar of the joint velocities (*ρ*) when Δ*m* was −1% (black dot-dashed line), −0.6% (red dashed line) and −0.2% (blue solid line).

To verify the robot had different motor capabilities with respect to *ρ* on the x-axis, we changed Δ*m*, fixed *D* (*i.e., D* = 0.0508,0.1016,0.2032 or 0.4064) and compared the quadratic coefficients among these various Δ*m's*.Figure 7 shows the quadratic coefficients when Δ*m* was -1%, -0.6% and -0.2% in each sub-figure, where (a), (b), (c) and (d) represent *D* = 0.0508, 0.1016, 0.2032 or 0.4064. We found that the robot with Δ*m* = -0.2% had the largest *pI_px_* compared to Δ*m* = -1% and -0.6%. In other words, the robot with the smaller error between actual and computed masses had the better motor capability. The simulation results validated Equation (14) because $\left(I_{x}^{T}{\text{J}}_{d}(\theta )K{\dot{\Phi }}_{0}^{T}\Delta \text{H}(\theta ){\dot{\Theta }}_{0}\right)$ and Δ***H***(***θ***) is definitely affected by Δ*m*.

### 4.2. Experiments on PUMA 560 and 260 Robots

**Figure 8.**
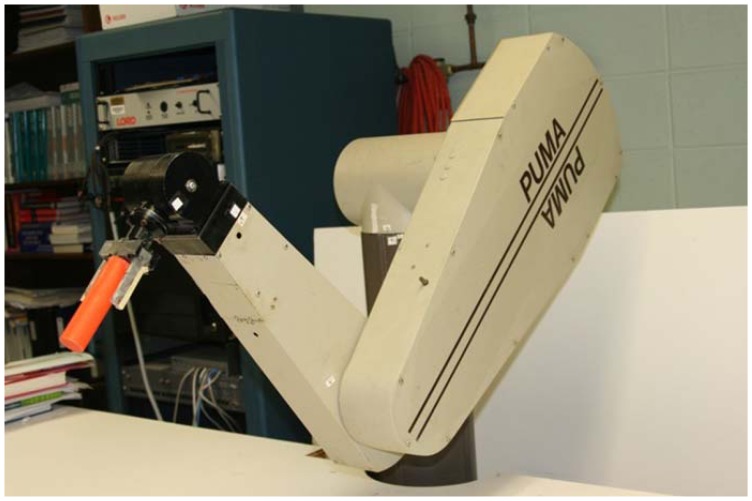
PUMA 560 robot in the experiment.

**Figure 9.**
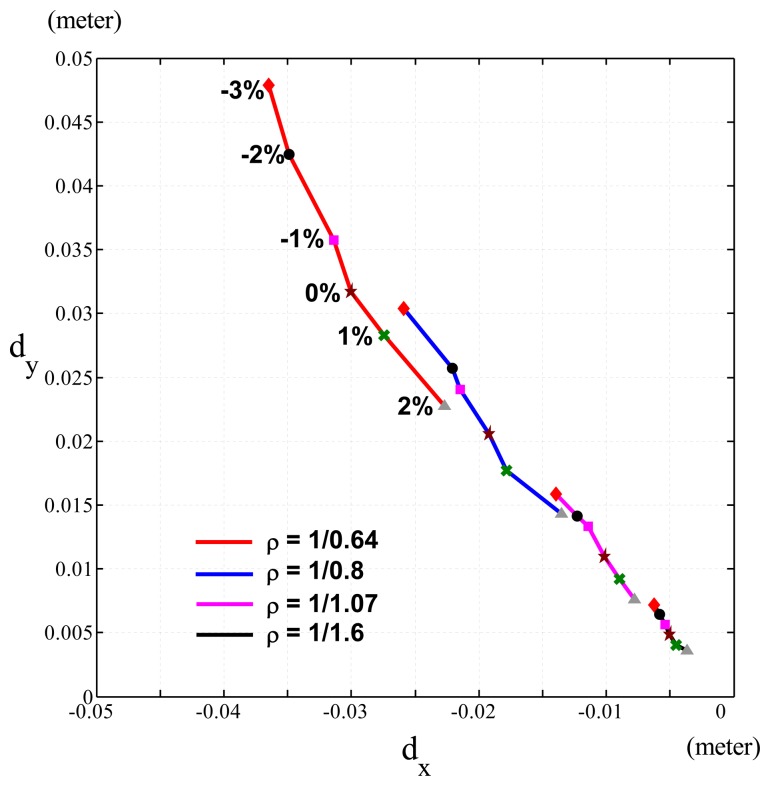
Experimental results of the Cartesian position errors of the end-effector caused by the changes of joint velocities (*ρ*) and computed masses, where ◆: Δ*m* = −3%; •: Δ*m* = −2%; ▪: Δ*m* = −1%; ★: Δ*m* = 0%; x: Δ*m* = 1%; ▲: Δ*m* = 2%.

To validate the speed-accuracy constraint, we performed the experiment on a PUMA 560 robot (see Figure 8). The joint distance, ***θ****_diff_*, between the initial and target positions of a joint-interpolated motion planning was [π/4,π/4,π/4,0,0,0], and the execution time for all the joints was set to *T_mt_* = 1.6,1.07,0.8 and 0.64 seconds for tests 1, 2, 3 and 4, respectively. Thus, the joint velocities of these tests were ***θ***(0304) = ***θ****_diff_/T_mt_* = *ρ* • ***θ***(0304)_0_(rad/sec), where ***θ***(0304)_0_ = ***θ****_diff_/*1 are nominal joint velocities finishing *θ_diff_* by one second and *ρ* = *1/* |*T_mt_*| = 0.625, 0.935,1.25,1.5625 for tests 1 to 4, respectively The relationships of joint velocities between test 1 and tests 2, 3 and 4 were obtained as ***θ̇***_2_ = 1.5***θ̇***_1_, ***θ̇****_3_* = 2***θ̇***_1_ and ***θ̇***_4_ = *2.5****θ̇***_1_. Although we could not obtain the actual masses of the links, we performed various Δ*m's* (the ratio of the error between the computed masses and the original computed masses we had to the original computed masses) by -3%, -2%, -1%, 0%, 1% and 2% and obtained similar results in Figure 5. Figure 9 shows the Cartesian position errors (*d_x_,d_y_*) of the end-effector of the PUMA 560 robot caused by the changes of joint velocities and Δ*m*. In Figure 9, each line was plotted by the Cartesian position errors of the end-effector of the robot introduced with various computed masses and the same joint velocity This shows that the Cartesian position error, ∝ Δ*m*, is under the same joint velocity

**Figure 10.**
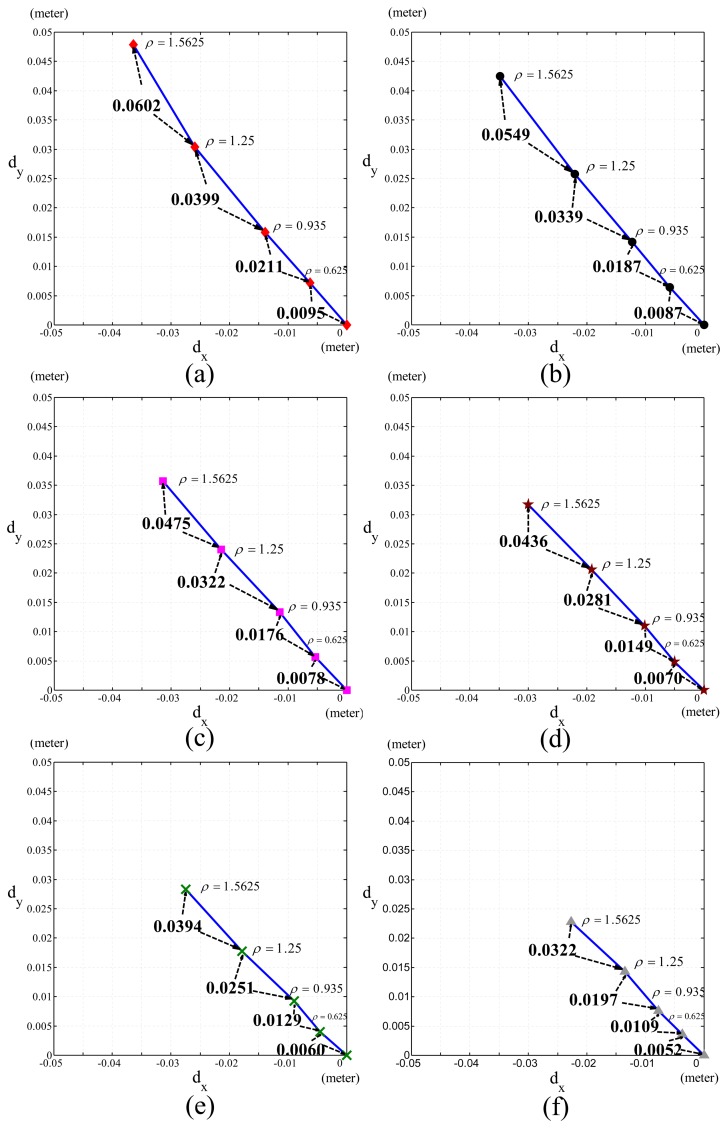
Experimental results of the Cartesian position errors of the end-effector caused by the fixed Am and the changes of the joint velocities (*ρ*). (a) Δ*m* = −3%; (b) Δ*m* = −2%; (c) Δ*m* = −1%; (d) Δ*m* = 0%; (e) Δ*m* = 1%; (f) Δ*m* = 2%.

From Figure 9, we obtain the relationship of Cartesian position errors caused by fixed Δ*m* and the changes of the joint velocities (*ρ*), as shown in Figure 10. Figure 10 represents Δ*m* for −3%, −2%, − 1%, 0%, 1% and 2%, respectively The ratios of the Cartesian position errors of the end-effector of the PUMA 560 robot caused by ***θ̇****_2_*,***θ̇***_3_ and ***θ̇***_4_ to the Cartesian position errors caused by ***θ̇***_1_ in Figure 10 were shown in Table 2. These ratios of errors were close to the expected ratios, *ρ*^2^ ((1.5)^2^ = 2.25, (2)^2^ = 4, (2.5)^2^ = 6.25), since ***θ̇***_2_ = 1.5***θ̇***_1_, ***θ̇***_3_ = *2****θ̇***_1_ and ***θ̇***_4_ = *2.5****θ̇***_1_. This shows that the Cartesian position error, ∝ *ρ*^2^, is under the same Δ*m*. Equation (13) explains this phenomenon when PUMA560 had a fixed *pI_px_*.

**Figure 11.**
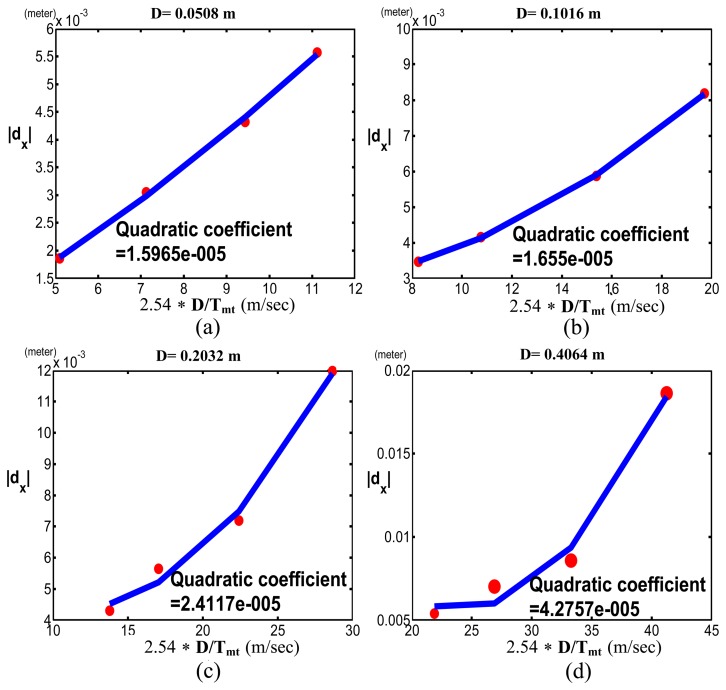
PUMA 560 experimental results of the Cartesian position errors of the end-effector along the *x*-axis (*d_x_*) with respect to *D/T_mt_* (**a**) D = 0.0508 m; (**b**) D = 0.1016 m; (**c**) D = 0.2032 m; (**d**) D = 0.4064 m.

**Figure 12.**
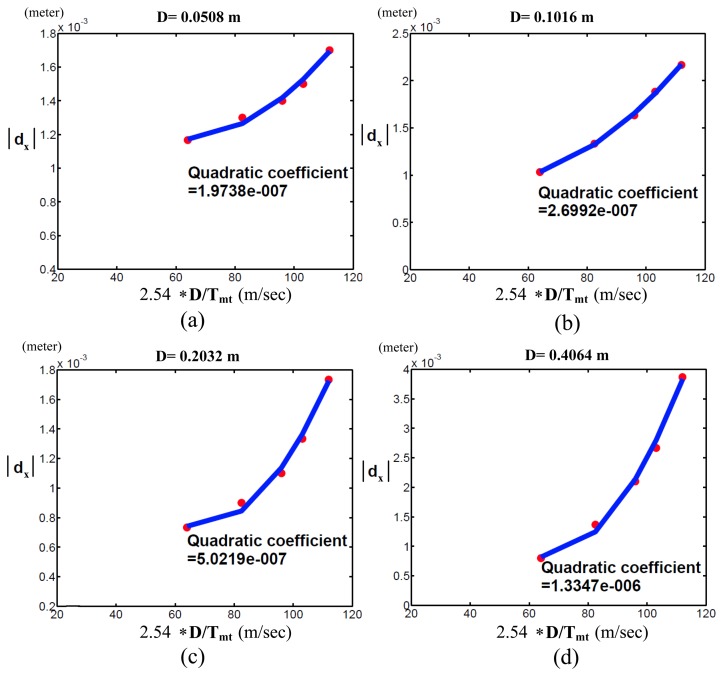
PUMA 260 experimental results of the Cartesian position errors of the end-effector along the *x*-axis (*d_x_*) with respect to *D/T_mt_* (**a**) D = 0.0508 m; (**b**) D = 0.1016 m; (**c**) D = 0.2032 m; (**d**) D = 0.4064 m.

To demonstrate that the pseudo-index of performance (*pI_px_*) is useful to evaluate the motor capability of various robots with revolute joints, we compared the motor capabilities of the PUMA 560 and 260 robots by measuring their *pI_px_*, where the PUMA 260 was a small robot with a similar mechanism to the PUMA 560. Figures 11 and 12 show the PUMA 560 and 260 robots' quadratic coefficients when *D* = 0.0508, 0.1016, 0.2032 and 0.4064. As *pI_px_* is the reciprocal of the quadratic coefficient, it is obvious that the PUMA 260 robot has better motor capability than the PUMA 560 robot.

## 5. Discussions and Conclusions

**Figure 13.**
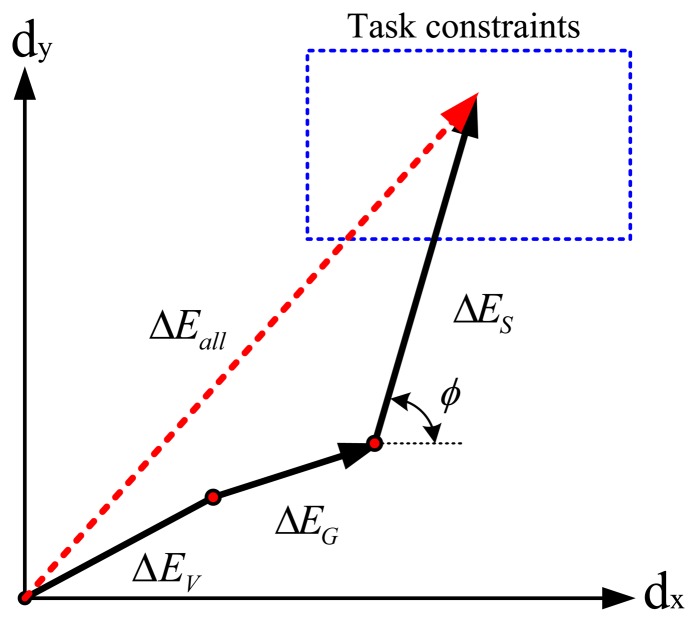
Vector representation of Cartesian position errors.

The proposed speed-accuracy constraints showed the relationship between Cartesian position errors of the end-effector of a robot and joint velocities. This relationship can be illustrated by a vector representation, as shown in Figure 13. For simplicity, Figure 13 shows the 2D projection of a 3D Cartesian position error caused by joint position errors (Δ***θ***). The Cartesian position error on the 2D plane, Δ*E_a_*_ll_, is composed of *d_x_* along the x-axis and *d_y_* along the *y*-axis. It is shown as the summation of Δ*E_V_*, Δ*E_G_* and Δ*E*_S_, where Δ*E_V_* represents the Cartesian position errors introduced by the errors between the actual and computed Coulomb frictions, Δ*E_G_* represents the Cartesian position errors introduced by the errors between the actual and computed gravity terms and Δ*E_S_* represents the Cartesian position errors regulated by the velocity changes. Here, Δ*E_G_* is affected by Δ*m* and Δ*E_G_*∝*α_m_*, where Δ*m* = *α_m_m* and *α_m_* is a scalar. Also, Δ*E_S_* is affected by θ and Δ*E_S_* ∝ *α_m_*. (*α_s_*)^2^, where ***θ̇*** = *α_s_****θ̇***_0_,***θ̇***_0_ is a reference speed and *α_s_* is a scalar. The angle, *φ*, is determined by *θ, m* and Δ*m*. When the desired joint positions, *θ*, and link masses, m and Am, are determined, we can choose *α_s_* (maximum speed) based on the vector relationship to make the vector, Δ*E_all_*, stay in the boundary of the task constraints.

From Equation (14), it is obviously shown that the pseudo-index of performance is the same with respect to the joint velocities, ***θ̇***, instead of the Cartesian speed, *dD/dt*. Thus, the pseudo-index of performance was not the same for the sub-figures of Figures 11 and 12. In addition, when a robot moves to a target in a fixed distance, Δ*m* of the robot will influence its motor capability. The less error in masses will give the robot more information capacity for trading off movement speed and accuracy to accomplish a task.

**Figure 14.**
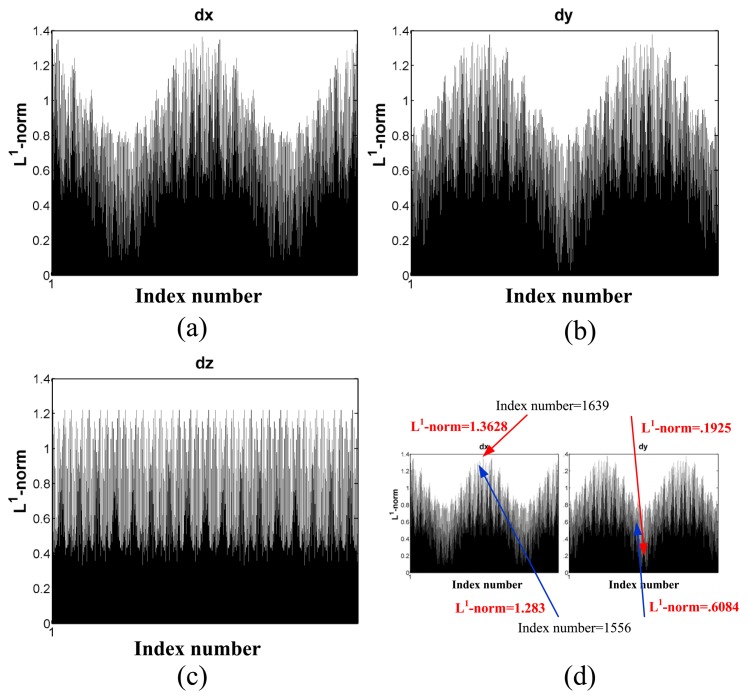
Cartesian position errors with respect to index number. Joint resolution is 20 degrees. (**a**) *d_x_*; (**b**) *d_y_*; (**c**) *d_z_*; (**d**) the same index number (joint positions) results in different *L*^1^-norm values on *d_x_* and *d_y_*.

The pseudo-index of performance is also affected by the target joint positions, ***θ***, because from Equation (14)***J****_d_*(***θ***) dramatically affects the Cartesian position errors, *d_x_, d_y_* and *d_z_*. Figure 14 shows the L^1^-norm of $I_{x}^{T}\cdot J_{d}(\theta )$, $I_{y}^{T}\cdot {\text{J}}_{d}(\theta )$ and $I_{z}^{T}\cdot J_{d}(\theta )$ with respect to index number, where ***I****_x_*,***I****_y_* and ***I****_z_* are unit vectors along the *x-, y*- and z-axis, respectively. L^1^-norm indicates the maximum Cartesian position errors, *d_x_, d*_y_ and *d_z_*, to which ***J****_d_*(***θ***) can contribute. The index number is generated from the data format as *index*_(_*_j_*_1)_:*index*_(_*_j2_*_)_:*index*_(_*_j3_*_)_:*index*_(_*_j4_*_)_:*index*_(_*_j5_*_)_:*index*_(_*_j6_*_)_, where *index*_(_*_ji_*_)_ is a sampling index by joint resolution within the joint range of joint, *i*[14], joint resolution is determined arbitrarily, *index*_(_*_j_*_1)_ are the most significant bits (MSB) and *index*_(_*_j6_*_)_ are the least significant bits (LSB). The index number represents the joint positions for all joints. To illustrate the concept and simplify the computation, joints 4, 5 and 6 are fixed as zero-degree, and the index number is only generated from joints 1, 2 and 3 with 20-degree joint resolution. Figure 14 shows the distribution of the *L*^1^-norm on *d_x_, d_y_* and *d_z_* with respect to the index number (joint positions). For some cases, an index number may have entirely different degrees of Cartesian position errors along the *x-, y*- and *z*-axis. For example, the index number, 1,639 (joint 1: 0 degree; joint 2: −125 degrees; joint 3: −45 degrees), has the maximum *L*^1^-norm on *d_x_*, but a relatively small *L*^1^-norm on *d_y_*. However, this index number is on the range boundary of joint 3, and it apparently is not an operation joint position. Similarly, another *L*^1^-norm on *d_x_* whose index number is 1,556 (joint 1: −20 degrees; joint 2: 35 degrees; joint 3: −25 degrees) is not on the joint range boundaries. For this index number, *d_x_* is also relatively larger than *d_y_*.

In this paper, we have developed and presented a quantitative measure, the pseudo-index of performance (*pI_p_*), to characterize the motor capability of a robot and the speed-accuracy constraints to optimize its motor performance for performing a given task. By using the *pI_p_*, one clearly understands the motor limitation of a robot. Since the pseudo-index of performance is based on the dynamics and kinematics of a robot system with revolute joints, the derived equations of the pseudo-index of performance are applicable to other robots with revolute joints. The PUMA 560 and 260 were revolute robots and feasibly the examples to validate the pseudo-index of performance. Computer simulations and experiments on PUMA 560 and 260 industrial robots have validated the characteristics of the speed-accuracy constraint and the *pI_p_* and how the *pI_p_* is utilized to measure the motor capability of a robot system.

## Figures and Tables

**Table 1.** Four movement velocities, D*/T_mt_* m/sec under different *D's*.

**D (m)**		***D/T****_mt_***(m/sec)**		
0.0508	0.0508/0.392	0.0508/0.281	0.0508/0.212	0.0508/0.180
0.1016	0.1016/0.484	0.1016/0.372	0.1016/0.260	0.1016/0.203
0.2032	0.2032/0.580	0.2032/0.469	0.2032/0.357	0.2032/0.279
0.4064	0.4064/0.731	0.4064/0.595	0.4064/0.481	0.4064/0.388

**Table 2.** Ratios of the Cartesian position errors of the end-effector of the PUMA 560 robot caused by ***θ̇***_2_,***θ̇***_3_ and ***θ̇***_4_ to the Cartesian position errors caused by ***θ̇***_1_.

**Δ*m***	***θ̇*_2_**	***θ̇*_3_**	***θ̇*_4_**
	*ρ*^2^ = 2.25	*ρ*^2^ = 4	*ρ*^2^ = 6.25
−3%	2.2115	4.1868	6.3067
−2%	2.1633	3.9134	6.3435
−1%	2.2556	4.1400	6.1096
0%	2.1339	4.0218	6.2426
1%	2.1253	4.1400	6.1096
2%	2.1066	3.8078	6.2284
